# Incomplete premating and postmating reproductive barriers between two parapatric populations of a social spider mite

**DOI:** 10.1007/s10493-015-9878-3

**Published:** 2015-01-30

**Authors:** Yukie Sato, Johannes A. J. Breeuwer, Martijn Egas, Maurice W. Sabelis

**Affiliations:** 1 Institute for Biodiversity and Ecosystem Dynamics, University of Amsterdam, P.O. Box 94240, 1090 GE Amsterdam, The Netherlands; 2 Laboratory of Animal Ecology, Department of Ecology and Systematics, Graduate School of Agriculture, Hokkaido University, Sapporo, 060-8589 Japan; 3 Sugadaira Montane Research Center, University of Tsukuba, Ueda, Nagano 386-2204 Japan

**Keywords:** Contact zone, Male–male conflict, Courtship behaviour, Mating behaviour, Reproductive isolation, Speciation, Acari, Tetranychidae

## Abstract

Closely related species with overlapping distributions often show premating reproductive barriers to avoid hybridization. *Stigmaeopsis miscanthi* (Saito) is a social spider mite infesting Chinese silver grass, and the species consists of two parapatric groups with frequent contacts within the contact zone. They differ in male–male aggressiveness, male morphology, female diapause traits, and life history parameters. There is incomplete but strong post-mating reproductive isolation between the two groups, and their DNA sequences are slightly different, suggesting that they diverged recently. In this study, we investigated premating reproductive barriers. We found that females from different groups frequently shared nest webs, indicating no barriers in the phase of nest establishment. However, inside nests, males from either group showed less courtship behaviour to females of the other group and they copulated less frequently with them when compared to females of the same group. However, the premating reproductive barrier was incomplete and asymmetric. Females of one group frequently resisted courtship by males from the other group, but females of the other group did not. We conclude that some gene flow may occur in the contact zone between the two groups.

## Introduction

Closely related species often show overlap in their geographic distribution. This geographic overlap may emerge during sympatric speciation, parapatric speciation or at secondary contact after allopatric speciation (Bull 1991). In each of these speciation scenarios, geographic barriers cannot prevent hybridization and one would expect premating reproductive barriers because premating reproductive barriers are more efficient at reducing the cost of hybridization (e.g. Servedio and Noor 2003; Coyne and Orr 2004).

*Stigmaeopsis* *miscanthi* (Saito) is a phytophagous mite on Chinese Silver grass, *Miscanthus sinensis* Andersson. In this species, the female mite weaves a silken nest on the undersurface of the leaves of their host plant. These nests are often shared with other females, males and offspring totalling more than one hundred individuals per nest (Saito 1997). These nests have a function as shelter (Mori et al. 1999; Horita et al. 2004), and the mites spend most of their lives inside nests. The adult females and males cooperatively defend their offspring against predators invading the nests (Yano et al. 2011). Males are also aggressive towards conspecific males, and they even kill males inside nests (Saito 1990). One explanation for this male–male aggression may be that it is relatively more effective to acquire immature females and mate with them just after they moult to adulthood rather than to look for other adult females available, because females primarily utilize sperm from the first mating and the second mating is not effective in spider mites (Boudreaux 1963; Helle 1967; Oku 2008). The degree of male–male aggression varies among populations in the mainland of Japan (Saito 1995): the males are highly aggressive in some populations (hereafter called HG = high in male aggression) and males show very little aggression in other populations (hereafter called LW = low in male aggression; Saito and Sahara 1999; Sato et al. 2013a). The aggression is correlated with the length of leg I relative to leg III, a measure of male weapon size (Saito 1995; Sato et al. 2013a). Subsequent studies revealed more differences between HG and LW populations: they differ in female diapause attributes (Saito et al. 2002) and life history traits (intrinsic rate of natural increase and developmental speed of juveniles; Saito et al. 2013). In addition, individuals from HG and LW populations appear to be genetically different. Ito and Fukuda (2009) showed that HG and LW have different mitochondrial COI haplotypes. They also appear to slightly differ at the nuclear 28S rDNA (Sakagami et al. 2009). Furthermore, compared to intra-population crosses, crosses between HG and LW populations display reduced juvenile survival rate and a large number of males with only occasionally a few females (Sato et al. 2000a, b). These characteristics indicate incomplete pre- and post-zygotic reproductive barriers, because this mite is haplodiploid: females develop from fertilized eggs whereas males develop from unfertilized eggs. Together, all these behavioural and genetic observations suggest that these populations have recently diverged.

**Fig. 1:**
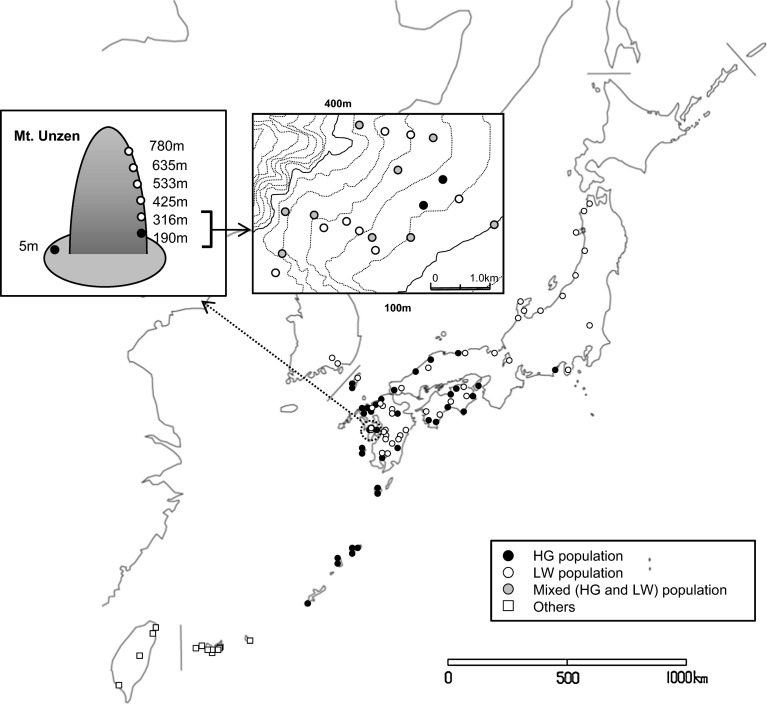
Localities where populations of *Stigmaeopsis miscanthi* were observed according to Saito and Sahara (1999), Sato et al. (2008) and Sato et al. (2013a). *Filled circles* indicate HG population, *open circles* indicate LW populations, *grey circles* indicate populations where HG and LW individuals were found together, and squares indicate populations which show intermediate male–male aggression. For details of populations shown by *grey circles*, see Sato et al. (2008). For details of population shown by *squares*, see Sato et al. (2013a)

The two groups are parapatrically distributed, with HG populations in warmer, southwestern regions (Shizuoka Prefecture to Okinawa Prefecture), and LW populations in cooler regions of all the large islands (except for Hokkaido and Ryukyu Islands) (Fig. 1). In southwest Japan (Shizuoka Prefecture to Kyushu Island), both groups are distributed as follows: HG populations in the lowlands, and LW populations in the highlands (Saito and Sahara 1999; Sato et al. 2013a). At intermediate elevation, their distributional ranges overlap and they coexist on the same grass stands (Sato et al. 2008; Fig. 1). Given that the two groups feed on the same host plant and their distributions overlap (Sato et al. 2008), the question arises how they reduce the costs from hybridization. One possibility is that premating reproductive barriers prevent hybridization between HG and LW groups. Mating in this mite species only takes place within nests (Sato et al. 2000a, b). So, one way to prevent mating between HG and LW is to share nests only with spider mites from the same group. To test this, we measured the probability of nest sharing between HG and LW females. Then, to determine if there is premating isolation between HG and LW, we measured the probability of individuals from the same group or from different groups to mate within nests. In addition, to determine at which points in time the courtship and/or mating behaviour is disturbed in crosses between HG and LW, we compared courtship and mating behaviour between HG and LW populations and between intra- and inter-population crosses.

## Materials and methods

### Female nest sharing

In 2009, we collected 30 HG females from a population in Tobuko (9 m above sea level) and 30 LW females from a population in Unzen (576 m above sea level) to initiate laboratory populations. The two populations were characterized as HG and LW based on an assessment of male–male aggression and male weapon size (Unzen = Ky 6 and Tobuko = Ky 7 in Sato et al. 2013a). Tobuko and Unzen are located in the same prefecture in Japan (Nagasaki Prefecture) and the distance between them is less than 10 km. In the laboratory, populations were maintained on detached leaves of the host plant (*M. sinensis* Andersson) on water-soaked cotton in Petri dishes under controlled conditions of 23 ± 2 °C, 40–70 % r.h. and a 15: 9 light: dark cycle.

To determine the probability that HG and LW females share nests, we introduced two females from the same or different populations on a piece of leaf in an arena for observation. The observation arena consisted of a 1.0 × 2.0 cm leaf piece cut from an intact *M. sinensis* leaf and placed on water-soaked cotton wool in a Petri dish. The edges of the leaf pieces were covered by thin strips of wet cotton wool that served as a barrier. Adult females were collected from laboratory populations without controlling for age and virginity and then they were introduced in the observation arena. One day after introduction, we recorded whether females were present in the same or another nest. If one or both females were missing in the arena due to drowning into the water-soaked cotton, the observation was discarded from further analysis. Because the physical space may affect a female’s choice to share a nest, we carried out the same experiment by using a 1.0 × 6.0 cm leaf piece from an intact *M. sinensis* leaf as the observation arena instead of a 1.0 × 2.0 cm leaf piece. Each possible combination of females was replicated 60 times, and the trials were conducted in random order.

We analyzed the probability that females shared a nest using a generalized linear model (GLM) with binomial error distribution taking female combination (HG pairs, LW pairs or mix) and the size of leaf arena (small or large) as explanatory variables. We tested the effect of explanatory variables by comparing the model with the explanatory variable to the model without the explanatory variable. In the model comparisons, we used a likelihood ratio test. All statistical analyses were done using the statistical package R, version 2.14. 2 (R Development Core Team 2012).

### Courtship and mating behaviour

The courtship and mating experiments were conducted between July and October 2003. Mites used in the experiments were collected in 2001 at the same locations as the nest sharing experiments but at slightly different elevation: Tobuko (59 m above sea level) and Unzen (780 m above sea level). The populations were characterized as HG or LW by measuring male weapon size (Sato et al. 2008). Each laboratory population was initiated with 30 females and maintained under the same conditions as described above.

**Fig. 2:**
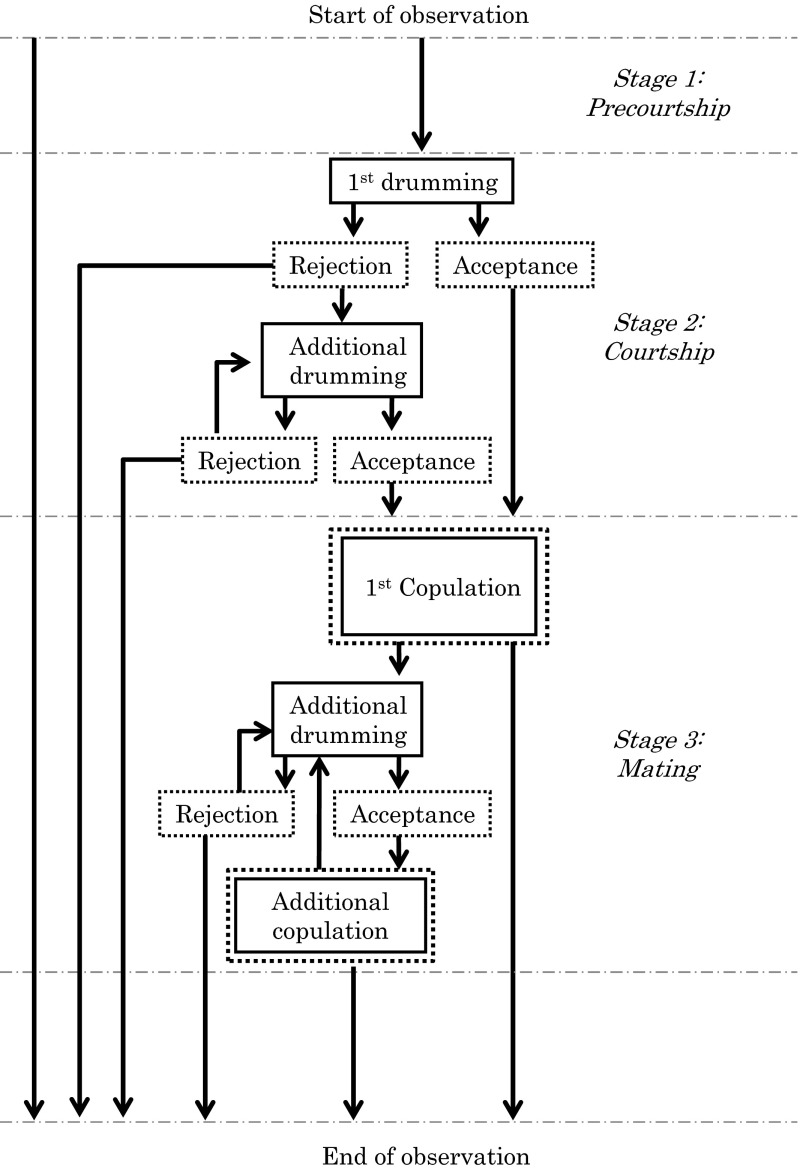
Sequence of events for the courtship and mating behaviour of *Stigmaeopsis miscanthi*. Male mating behaviours are shown in *boxed text* and female mating behaviours are shown in *dashed boxed* *text*. Arrows show the transition of behaviours. The sequence of events is distinguished into three stages: precourtship, courtship, and mating. Precourtship stage is from the male introduction (start of observation) to the point that the male started the first drumming event. Courtship stage is from the first drumming event to the point that the female raised her abdomen. Mating stage is from the first copulation to the point that the male stopped copulating or drumming

To determine if there is premating isolation between HG and LW individuals, courtship and mating behaviour was observed in single-pair crosses between HG and LW populations. Single-pair crosses between a virgin female and a virgin male were set up in the following way. Because this mite has haplodiploid sex determination, virgin females produce only sons, whereas mated females produce both sons and daughters and the offspring sex ratio is biased toward females. Hence, in this experiment, virgin males were obtained from virgin mothers. Virgin females were obtained by collecting them as teleiochrysalis (last moulting stage) from the cultures. For each cross, a teleiochrysalis female was placed in a small observation arena (1.0 × 2.0 cm leaf piece). After moulting, the adult female was allowed to construct a nest within 24 h. If the nest was constructed, a 1–5 day old virgin male was introduced to the female in the observation arena. If no nest was constructed, the female was discarded. The courtship and mating behaviour was videotaped for 1 h after introduction of the male. We assessed the number of pairs in which copulation was observed. Afterwards, we distinguished three stages in the possible sequence of events: precourtship, courtship, and mating (see Fig. 2). To determine at which points in time the courtship and mating behaviour was disturbed in crosses between HG and LW, we measured the transition probabilities from precourtship to courtship, identified by the start of the first drumming event (behaviour of the male involving tapping the dorsal part of the female’s abdomen with his first pair of legs while extending his aedeagus), and from courtship to mating, identified by the moment the female raises her abdomen. We also measured the following behavioural traits: duration of the precourtship stage (which is the time between male introduction and the first drumming event; see Fig. 2), duration of the first drumming event, duration of the first and additional copulations, the number of drumming events prior to the first copulation, and the number of copulations within the observation period. To check postmating reproductive barriers in these single-pair crosses, males were removed after 1 h observation and females were allowed to oviposit for 10 days. Then, the number of eggs, juvenile survival rate and offspring sex ratios were determined as measures for postmating barriers. Each possible combination of male and female was replicated 20–25 times in the trials using HG males and 25–30 times in the trials using LW males, since we found that LW males copulate less frequently within 1 h. The trials were conducted in random order.

The probability that the pairs copulated and the transition probability from precourtship to courtship were analyzed using GLMs taking cross type (intra- or inter-population) and male type (HG or LW) as explanatory variables. The transition probability from courtship to mating was analyzed using a GLM taking cross type and female type (HG or LW) as explanatory variables.

Courtship and mating behaviour was compared in intra-population crosses at first, by analyzing the following variables: duration of precourtship stages, duration of the first drumming event, the number of drumming events, the number of copulations, duration of the first copulations and total duration of copulations (sum of durations of the first and additional copulations). Duration of precourtship stages, the number of drumming events and the number of copulations were analyzed using the Wilcoxon rank sum test. Duration of the first drumming event was analyzed using a GLM taking male type and the outcome of the drumming events (whether or not the females accepted the drumming action and copulated) as explanatory variables and assuming a gamma distribution as the error distribution. Duration of the first copulations and total duration of copulations were analyzed using Welch two sample *t* test.

Next, courtship and mating behaviour was compared between intra- and inter-population crosses by using the same variables. All variables, except for duration of the first drumming event, were analyzed using GLMs taking crossing type and male type as explanatory variables. Duration of the first drumming event was analyzed for each male type using GLMs taking crossing type and outcome of the drumming event as explanatory variables. This was done because LW males extended the time spent drumming when females did not accept them as mating partners whereas HG males’ drumming were constant regardless of the females’ response to the drumming actions. The number of eggs, juvenile survival rate and offspring sex ratios (female ratios) were analyzed using GLMs taking cross type and female type as explanatory variables.

In the analyses using GLMs, we used a binomial error distribution in case the response variable represented a probability, a Poisson error distribution in case the response variable was a number, and a Gaussian or gamma error distribution in case the response variable was based on duration. When we detected overdispersion in the GLM, we assumed a quasi-binomial or a quasi-Poisson error distribution instead of a binomial or Poisson error distribution. The effect of each explanatory variable was tested by comparing the model with the explanatory variable to the model without the explanatory variable using a likelihood ratio test. All statistical analyses were done using the statistical package R, version 2.14. 2 (R Development Core Team 2012).

## Results

### Female nest sharing

**Fig. 3:**
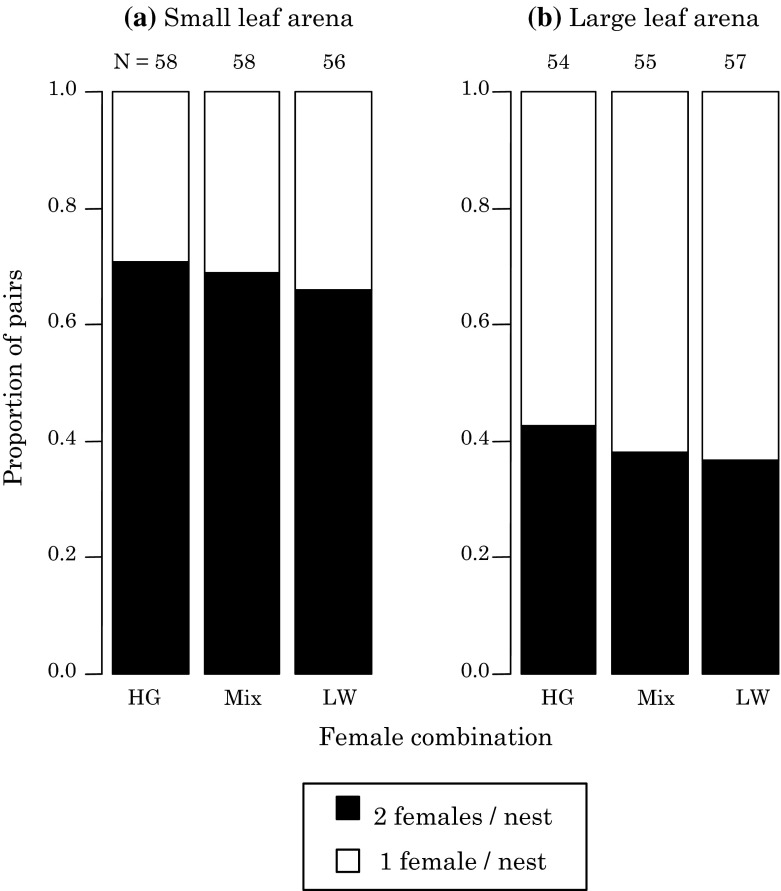
Proportions of pairs of *Stigmaeopsis miscanthi* in which two females from the same or different groups construct nests together when they were introduced on a small leaf arena (**a**) and a large leaf arena (**b**) in the experiment of female nest sharing

The probability that females shared a nest in small arenas was 0.6–0.7 and significantly higher than that in large arenas (0.35–0.45) (Fig. 3; *Χ* ^2^ = 29.843, *df* = 1, *P* < 0.001). There was no significant difference in nest sharing between intra-population and inter-population combinations of females (*Χ* ^2^ = 0.668, *df* = 2, *P* = 0.72).

### Courtship and mating behaviour

All males (n = 103) immediately entered the nests after introduction into the arena regardless of the female type who constructed the nests. After entering the nest and physical contact with a female, the male courted the female by drumming the dorsal side of her abdomen with his first pair of legs while extending his aedeagus (Fig. 2). When the female lifted her abdomen and exposed her genital orifice, the male maneuvered under the female, curled up his abdomen and copulated with the female by extending his aedeagus into the female genital orifice.

The probability that a pair copulated was assessed from observations (Table 1). It was significantly lower in the inter-population than intra-population crosses (*Χ* ^2^ = 9.020, *df* = 1, *P* = 0.003). In addition, LW males were less likely to copulate compared to HG males (*Χ* ^2^ = 18.110, *df* = 1, *P* < 0.001). Next, we analyzed transition probabilities from one stage to the next and behavioural traits to determine at which points in time the courtship and mating behaviour was disturbed in inter-population crosses.

**Table 1:** Transition probabilities from precourtship to courtship stages and from courtship to mating stages, percentage of pairs in which copulation was observed, in intra- and inter-population crosses of *Stigmaeopsis miscanthi*

Cross type	Female	Male	No. of pairs	Transition: Precourtship stage → Courtship stage	No. of courting pairs	Transition: Courtship stage → Mating stage	No. of copulating pairs	Percent copulating pairs
Intra-population	HG	HG	20	1.00	20	1.00	20	100
	LW	LW	29	0.83	24	0.83	20	69.0
Inter-population	HG	LW	30	0.57	17	0.59	10	33.3
	LW	HG	24	0.92	22	0.91	20	83.3

For the details of precourtship, courtship and mating stages, see the sequence of events for the mating behaviour in Fig. 2

#### Precourtship to courtship stage

The transition probability from precourtship to courtship, as identified by the start of the first drumming event, was significantly higher in intra-population crosses compared to inter-population crosses (Table 1; *Χ* ^2^ = 6.551, *df* = 1, *P* = 0.010). In intra-population crosses, all HG males and the majority (82.8 %) of LW males showed drumming behaviour, whereas in inter-population crosses, 91.7 % of HG males and 56.7 % of LW males showed drumming behaviour. In general, drumming behaviour was significantly less frequent in LW males compared to HG males (Table 2; *Χ* ^2^ = 13.803, *df* = 1, *P* < 0.001).

**Table 2:** The number of drumming events in the courtship stage and the number of copulations in mating stage observed in intra- and inter-population crosses of *Stigmaeopsis miscanthi*

Cross type	Female	Male	No. of courting pairs	No. of drum sessions (mean ± SE)	No. of copulating pairs	No. of copulations (mean ± SE)
Intra-population	HG	HG	20	1.35 ± 0.15	20	2.20 ± 0.42
	LW	LW	24	1.25 ± 0.12	20	1.50 ± 0.20
Inter-population	HG	LW	17	1.27 ± 0.20	10	1.60 ± 0.27
	LW	HG	22	1.35 ± 0.11	20	2.55 ± 0.34

For the details of courtship and mating stages, see the sequence of events for the mating behaviour in Fig. 2

**Fig. 4:**
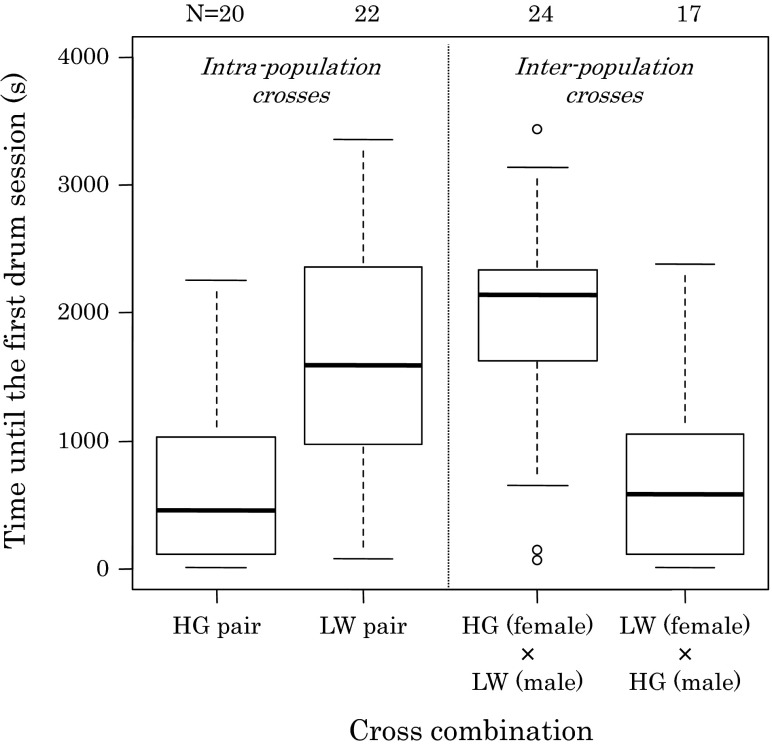
*Box plots* of durations of precourtship stage (duration time until males showed courtship behaviour to females) in intra- and inter-population crosses in the experiment of courtship and mating behaviour of *Stigmaeopsis miscanthi*

**Fig. 5:**
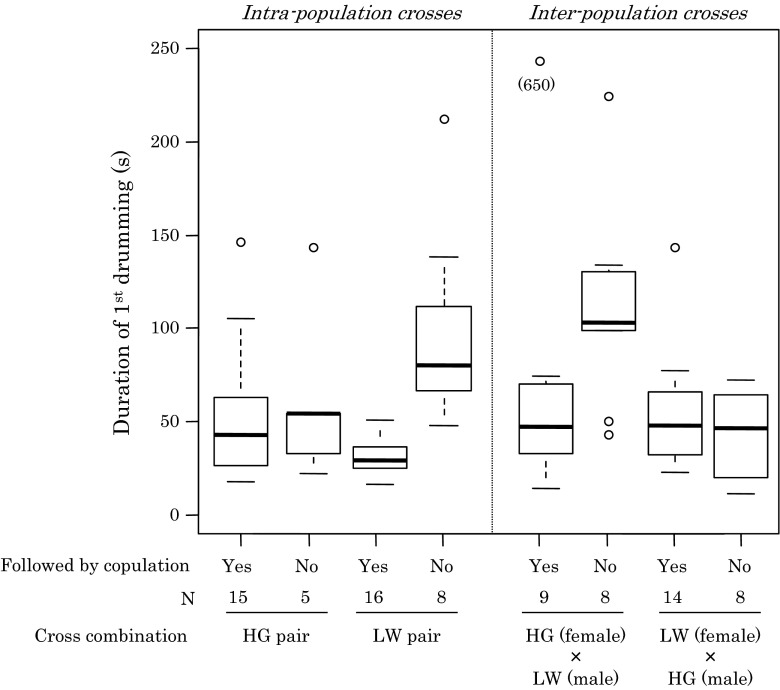
*Box plots* of durations of the first drumming events followed by copulations (successful drumming events) and not followed by copulations (unsuccessful drumming events) in intra- and inter-population crosses in the experiment of courtship and mating behaviour of *Stigmaeopsis miscanthi*

In intra-population crosses, HG males started drumming after 655 ± 139 s (mean ± SE) from the start of observation, which was significantly faster than LW males who started courtship after 1,645 ± 192 s (Fig. 4; *U* = 86, *P* < 0.001). Duration of the first drumming event, only including drumming that was followed by copulation, was not significantly different between HG males (53 ± 9.4 s) and LW males (31 ± 2.5 s) (Fig. 5; *U* = 162, *P* = 0.099). Duration of the first drumming event was affected by whether or not it was followed by copulation in LW males, but not in HG males (Fig. 5; *F* = 9.950, *df* = 1, *P* = 0.003). The duration of unsuccessful drumming of LW males (97 ± 19 s) was significantly longer than successful drumming (31 ± 2.5 s). The number of drumming events before moving to the mating phase did not significantly differ between HG couples (1.35 ± 0.15 times), and LW couples (1.25 ± 0.12 times) (*U* = 249, *P* = 0.90), and varied from one to three drumming events (Table 2).

Next, we compared courtship and mating behaviour between inter- and intra-population crosses. There were no significant differences between inter- and intra-population crosses in duration of the precourtship phase (Fig. 4; cross type: *F* = 0.276, *df* = 1, *P* = 0.60; male type: *F* = 16.189, *df* = 1, *P* < 0.001), the number of drumming events (Table 2; cross type: *Χ* ^2^ = 0.001, *df* = 1, *P* = 0.97; male type: *Χ* ^2^ = 0.107, *df* = 1, *P* = 0.74), and the duration of the first drumming event in HG males (Fig. 5; cross type: *F* = 0.230, *df* = 1, *P* = 0.64; result of drum session (copulation followed or not): *F* = 0.057, *df* = 1, *P* = 0.81) and in LW males (cross type: *F* = 1.584, *df* = 1, *P* = 0.22; outcome of drumming (copulation followed or not): *F* = 51.131, *df* = 1, *P* < 0.001; note that this analysis was performed by removing one extreme outlier [i.e. 650 s) in HG (female) × LW (male)].

#### Courtship to mating stage

The probability of copulation given that the males had started a drumming event (transition probability from stage 2 to 3 in Table 2) was significantly higher in intra-population compared to inter-population crosses (*Χ* ^2^ = 4.803, *df* = 1, *P* = 0.028). Overall, LW males were less likely to copulate compared to HG males (*Χ* ^2^ = 9.979, *df* = 1, *P* = 0.002).

**Fig. 6:**
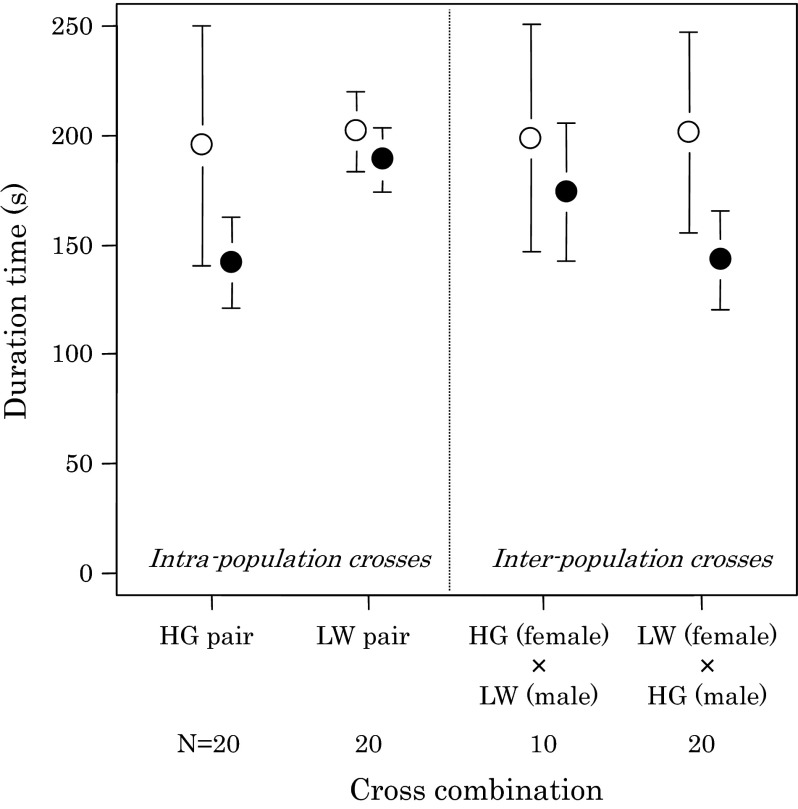
Duration of copulations observed in intra- and inter-population crosses of *Stigmaeopsis miscanthi*. Multi-copulation within a pair was observed (Table 1): the first copulation and additional copulations, which were much shorter than the first copulation. *Filled circles* indicate the average durations of the first copulations, and *open circles* indicate the average total durations of copulations (the sum of durations of the first and additional copulations). *Vertical bars* indicate 95 % confidence interval

The number of copulations was 2.2 ± 0.4 in HG couples and 1.5 ± 0.2 in LW couples, and varied from one to 8 copulations (Table 2). The difference in the number of copulations between them was not significant (*U* = 243, *P* = 0.20). Duration of the first copulations in HG couples (142 ± 10 s) was significantly shorter than that in LW couples (189 ± 07 s) (Fig. 6; *t* = −3.834, *df* = 33.932, *P* < 0.001). Total duration of copulations observed in the mating stage was not significantly different between HG and LW couples (Fig. 6; *t* = −0.239, *df* = 23.099, *P* = 0.81).

Next, we compared mating behaviour between inter- and intra-population crosses. There were no significant differences in the number of copulations (Table 2; cross type: *Χ* ^2^ = 0.509, *df* = 1, *P* = 0.48; male type: *Χ* ^2^ = 5.468, *df* = 1, *P* = 0.019), duration of the first copulations (Fig. 6; cross type: *F* = 0.259, *df* = 1, *P* = 0.61; male type: *F* = 15.384, *df* = 1, *P* < 0.001), and the sum of duration of the first and additional copulations (cross type: *F* = 0.013, *df* = 1, *P* = 0.91; male type: *F* = 0.017, *df* = 1, *P* = 0.90).

#### Postmating reproductive barriers

There was no significant difference between intra- and inter-population crosses in the number of eggs per female produced over a period of 10 days (Table 3; cross type: *Χ* ^2^ = 0.173, *df* = 1, *P* = 0.68; female type: *Χ* ^2^ = 1.302, *df* = 1, *P* = 0.25). Juvenile survival rate was significantly lower in inter-population crosses compared to intra-population crosses (Table 3; cross type: *F* = 4.596, *df* = 1, *P* = 0.038; female type: *F* = 5.907, *df* = 1, *P* = 0.019). Offspring sex ratio (female proportion) was also significantly lower in inter-population crosses compared to intra-population crosses (Table 3; cross type: *Χ* ^2^ = 260.360, *df* = 1, *P* < 0.001; female type: *Χ* ^2^ = 0.461, *df* = 1, *P* = 0.50).

**Table 3:** The number of eggs per female laid in 10 days, juvenile survival rate of the offspring (from egg to adult) and proportion of females in offspring observed in the experiment of courtship and mating behaviour of *Stigmaeopsis miscanthi*

Cross type	Female	Male	No. of pairs	No. of eggs (mean ± SE)	Juvenile survival rate (mean ± SE)	Offspring sex ratio (mean ± SE)
Intra-population	HG	HG	12	11.42 ± 0.91	0.97 ± 0.02	0.87 ± 0.01
	LW	LW	15	12.40 ± 0.69	0.93 ± 0.02	0.86 ± 0.01
Inter-population	HG	LW	7	10.71 ± 1.23	0.95 ± 0.03	0.14 ± 0.04
	LW	HG	13	12.15 ± 0.88	0.82 ± 0.05	0.22 ± 0.05

## Discussion

Two different sets of experiments served to investigate the degree of premating reproductive isolation between high male aggression (HG) and low male aggression populations (LW). The first set of experiments investigated the probability that females of the two different populations shared a nest. There was no difference in the probability sharing a nest between females from the same and different populations, although the size of leaf arena had an effect on the frequency of female nest sharing (Fig. 3). Males of either population did not discriminate between the nests constructed by females from the same group and those by females from another group, and frequently engaged in fights with males from another group to gain nest ownership (Sato et al. 2013b). These results suggest that nest sharing does not act as a reproductive barrier because females and males easily share the nest with mites from another group whereas mating takes place within nests, thereby enabling mixed offspring to emerge.

The second set of experiments investigated courtship and mating behaviour. Both populations showed stereotypic courtship and mating behaviour of spider mites (Cone 1985), and consisted of a male drumming event followed by acceptance of the female and copulation. In general, LW couples were less likely to mate than HG couples in 1 h mating trials: not all LW males started drumming and not all LW females accepted drumming males (Table 1). Probably more LW crosses would have resulted in copulations, if couples were given more time in the mating trials. Indeed, the precourtship phase of LW males lasted two to three times longer than that of HG males (Fig. 4). In more than half of the LW × LW replicates, it required more than 44 % (median = 1,590 s) of the observation period of 3,600 s (1 h). Strikingly, LW males were more persistent in drumming than HG males. They did not end the drumming as quickly as HG males when a female was not responsive (Fig. 5). The persistent drumming of LW males was not only observed in intra-population crosses but also inter-population crosses. Although total time *in* *copula* in intra-population crosses was not different (Fig. 6), the first copulation of LW males lasted longer compared to HG males (Fig. 6), because HG males were more likely to copulate multiple times compared to LW males (Table 1). These differences in courtship and mating behaviour are possibly linked to differences in male–male aggression, suggesting a behavioural syndrome (suites of correlated behaviours across different contexts and situations; Sih et al. 2004).

Comparing mating behaviour between intra- and inter-population crosses, the percentage of pairs copulating within 1 h mating trials was significantly lower in inter-population crosses (Table 1). Although the duration of the precourtship stage was not different between intra- and inter-population crosses (Fig. 4), males were more likely to start drumming the dorsum of females from the same population than females from the other population (Table 1). This suggests that females from different groups are less attractive compared to females from the same group. Although it appears that females were less likely to accept copulations after their dorsum was drummed by males from another population (Table 1), this was not significant.

Taking all data together, a premating barrier between HG and LW exists. However, the barrier is incomplete and asymmetric: males showed less preference to females from another group and HG females less frequently accepted courtship by LW males, whereas LW females accepted the courtship by HG males more frequently (Table 1). Since premating as well as postmating reproductive barriers were incomplete between HG and LW, multiple isolating barriers are required to limit the production of hybrid offspring, as observed in many animals (e.g. Coyne and Orr 2004; Matsubayashi and Katakura 2009; Scopece et al. 2013). Yet, despite the limited production of hybrid offspring due to these combined barriers (Table 2; Sato et al. 2000a, b), there is a possibility for gene flow in the contact zone. Indeed, hybrids, especially produced by LW (female) × HG (male), have reproductive ability (Sato, unpublished data), and males with intermediate morphology between HG and LW have been found in the field in the area where HG and LW populations overlap (Sato et al. 2008). Hence, the genetic differences between HG and LW populations may be maintained not only by reproductive incompatibility but also by other factors, such as natural selection against hybrids in either area where the HG or LW population occurs. However, it is also possible that the genetic differences are not strongly maintained. Male-male aggression varies not only between HG and LW groups but also between populations within an HG group and within an LW group. The geographic variation in male–male aggression is explained by kin selection: male–male aggression is high in kin-groups and relatively low in non-kin groups (Saito 1995; Saito and Sahara 1999; but see also Sato et al. 2013a). However, the geographic variations observed within either of the two groups can be caused by gene flow between HG and LW groups. To test these hypotheses, population genetic analyses would be required.

In this study, we mainly focus on how the two populations reduce the cost from hybridization. However, to understand their speciation process, it is also important how the two populations maintain their parapatric distribution. Sato et al. (2008) proposed that LW populations may not be displaced by HG populations, because LW has a more intense diapause than HG (Saito et al. 2002). If HG expands its range into cooler regions (highlands), HG would fail to overwinter in the region because of its diapause attributes. Conversely, HG populations would not be displaced by LW populations either, because if LW expands its range into warmer regions (lowlands), LW females possibly suffer from reproductive interference by HG males. The latter part was supported by the relationship between HG and LW males: HG and LW males fight for ownership of nests, and HG males often win the fights (Sato et al. 2013b). Moreover, the findings of our study are in support: LW females mate with HG males more frequently than with LW males, whereas HG females rarely mate with LW males (Table 1). Asymmetric mate preference between closely related species has been found in many species including mites, and they are frequently discussed with consideration of reproductive interference and their spatial and temporal segregation (e.g. Takafuji et al. 1997; Hochkirch et al. 2007; Noriyuki et al. 2012; Sato et al. 2014). Parapatric distribution of the two mite groups would represent a case where asymmetric mate preference has an impact on the spatial segregation between two closely related species.

In our study, we also found a possibility for a behavioural syndrome: males with higher male–male aggression show higher activity in mating behaviour. Recent work focuses on behavioural syndromes in animals other than rodents and primates (e.g., reviewed by Sih et al. 2004). For example, in the field cricket, male–male aggression over females and over territories correlates with latencies to become active when placed in a novel environment and latencies to emerge from safe refuge (Kortet and Hedrick 2007). However, in *Drosophila* *melanogaster*, selection for aggressive behaviour did not change the male courtship behaviour, mating duration, and latency to mating (Dierick and Greenspan 2006). Behavioural syndromes are a relatively novel concept in behavioural ecology of invertebrates, therefore, more research is required to understand how common it is and how it evolves. Considering that male–male aggression varies not only between the groups but also between populations within each group in the mite species (Saito and Sahara 1999; Sato et al. 2013a), it would be worthwhile to investigate the correlation between male–male aggression and male mating activity. If such correlation exists, the resulting premating barrier would vary depending on the pairs of HG and LW populations. Although we used one HG population and one LW population in our study, research using various populations different in male–male aggression would be required to investigate the behavioural syndrome in this mite species and also to investigate variation in intensity of premating barriers between groups.

## References

[CR1] Boudreaux HB (1963). Biological aspects of some phytophagous mites. Annu Rev Entomol.

[CR2] Bull CM (1991). Ecology of parapatric distributions. Annu Rev Ecol Syst.

[CR3] Cone WW (1985) Mating and chemical communication. In: Helle W, Sabelis MW (eds) Spider mites: Their. biology, natural enemies and control, vol 1A. Elsevier, Amsterdam, pp 243–251

[CR4] Coyne JA, Orr HA (2004). Speciation.

[CR5] Dierick HA, Greenspan RJ (2006). Molecular analysis of flies selected for aggressive behavior. Nat Genet.

[CR6] Helle W (1967). Fertilization in the two-spotted spider mite (*Tetranychus urticae*: Acari). Entomol Exp Appl.

[CR7] Hochkirch A, Gröning J, Büker A (2007). Sympatry with the devil: reproductive interference could hamper species coexistence. J Anim Ecol.

[CR8] Horita M, Chittenden AR, Sato Y, Saito Y (2004). Function of the web box as an anti-predator barrier in the spider mite, *Schizotetranychus recki*. J Ethol.

[CR9] Ito K, Fukuda T (2009). Molecular phylogeny of *Stigmaeopsis* spider mites (Acari: Tetranychidae) based on the cytochrome oxidase subunit I (COI) region of mitochondrial DNA. Appl Entomol Zool.

[CR10] Matsubayashi KW, Katakura H (2009). Contribution of multiple isolating barriers to reproductive isolation between a pair of phytophagous lady bird beetles. Evolution.

[CR11] Mori K, Saito Y, Sakagami T (1999). Effects of the nest web and female attendance on survival of young in the subsocial spider mite *Shizotetranychus longus* (Acari: Tetranychidae). Exp Appl Acarol.

[CR12] Noriyuki S, Osawa N, Nishida T (2012). Asymmetric reproductive interference between specialist and generalist predatory ladybirds. J Anim Ecol.

[CR13] Oku K (2008). Is only the first mating effective for females in the Kanzawa spider mite, *Tetranychus kanzawai* (Acari: Tetranychidae)?. Exp Appl Acarol.

[CR31] R Development Core Team (2012) R: a language and environment for statistical computing. R Foundation for Statistical Computing, Vienna, Austria. http://www.R-project.org/

[CR14] Saito Y (1990). ‘Harem’ and ‘non-harem’ type mating systems in two species of subsocial spider mites (Acari, Tetranychidae). Res Popul Ecol.

[CR15] Saito Y (1995). Clinal variation in male-to-male antagonism and weaponry in a subsocial mite. Evolution.

[CR16] Saito Y, Choe JC, Crespi BJ (1997). Sociality and kin selection in Acari. The evolution of social behavior in insects and arachnids.

[CR17] Saito Y, Sahara K (1999). Two clinal trends in male-male aggressiveness in a subsocial spider mite (*Schizotetranychus miscanthi*). Behav Ecol Sociobiol.

[CR18] Saito Y, Sakagami T, Sahara K (2002). Differences in diapause attributes between two clinal forms distinguished by male-to-male aggression in a subsocial spider mite, *Schizotetranychus miscanthi* Saito. Ecol Res.

[CR19] Saito Y, Kanazawa M, Sato Y (2013). Life history differences between two forms of the social spider mite, *Stigmaeopsis miscanthi*. Exp Appl Acarol.

[CR20] Sakagami T, Saito Y, Kongchuensin M, Sahara K (2009). Molecular phylogeny of *Stigmaeopsis*, with special reference to speciation through host plant shift. Ann Entomol Soc Am.

[CR21] Sato Y, Saito Y, Mori K (2000). Reproductive isolation between populations showing different aggression in a subsocial spider mite, *Schizotetranychus miscanthi* Saito (Acari: Tetranychidae). Appl Entomol Zool.

[CR22] Sato Y, Saito Y, Mori K (2000). Patterns of reproductive isolation between two groups of *Schizotetranychus miscanthi* Saito (Acari: Tetranychidae) showing different male aggression traits. Appl Entomol Zool.

[CR23] Sato Y, Saito Y, Chittenden AR (2008). The parapatric distribution and contact zone of two forms showing different male-to-male aggressiveness in a social spider mite, *Stigmaeopsis miscanthi* (Saito) (Acari: Tetranychidae). Exp Appl Acarol.

[CR24] Sato Y, Egas M, Sabelis MW, Mochizuki A (2013). Male-male aggression peaks at intermediate relatedness in a social spider mite. Ecol Evol.

[CR25] Sato Y, Sabelis MW, Mochizuki A (2013). Asymmetry in male lethal fight between parapatric forms of a social spider mite. Exp Appl Acarol.

[CR26] Sato Y, Alba JM, Sabelis MW (2014). Testing for reproductive interference in the population dynamics of two congeneric species of herbivorous mites. Heredity.

[CR27] Scopece G, Croce A, Lexer C, Cozzolino S (2013). Components of reproductive isolation between *Orchis mascula* and *Orchis pauciflora*. Evolution.

[CR28] Servedio MR, Noor MAF (2003). The role of reinforcement in speciation: Theory and data. Annu Rev Ecol Evol Syst.

[CR29] Sih A, Bell A, Johnson JC (2004). Behavioral syndromes: an ecological and evolutionary overview. Trends Ecol Evol.

[CR30] Takafuji A, Kuno E, Fujimoto H (1997). Reproductive interference and its consequences for the competitive interactions between two closely related *Panonychus* spider mites. Exp Appl Acarol.

[CR32] Yano J, Saito Y, Chittenden AR, Sato Y (2011). Variation in counterattack effect against a phytoseiid predator between two forms of the social spider mite, *Stigmaeopsis miscanthi*. J Ethol.

